# Maternal and neonatal outcomes associated with induction of labor after one previous cesarean delivery: A French retrospective study

**DOI:** 10.1371/journal.pone.0237132

**Published:** 2020-08-07

**Authors:** Emma Vecchioli, Anne-Gaël Cordier, Anne Chantry, Alexandra Benachi, Isabelle Monier

**Affiliations:** 1 Department of Obstetrics and Gynaecology, AP-HP, Antoine Béclère Hospital, University Paris Saclay, Clamart, France; 2 Midwifery School of Baudelocque, Paris-Descartes University, AP-HP, DHU Risks in Pregnancy, Paris, France; 3 Obstetrical, Perinatal and Pediatric Epidemiology Research Team (EPOPé), Université de Paris, Epidemiology and Statistics Research Center (CRESS), INSERM, INRA, Paris, France; Federal University of Sergipe, BRAZIL

## Abstract

**Background:**

The safety of methods of labor induction in women with previous cesarean deliveries is still debated. We investigated perinatal outcomes associated with labor induction among women with a trial of labor after one cesarean delivery.

**Methods:**

This retrospective study included 339 women with a trial of labor after one prior cesarean and a singleton term fetus in cephalic presentation in 2013–2016 in a French maternity unit. Labor induction was performed with oxytocin, artificial rupture of membranes and/or prostaglandin E2, according to the Bishop score. The primary outcome was a composite of uterine rupture, low Apgar score, neonatal resuscitation or admission to a neonatal unit. The secondary outcomes included cesarean delivery after onset of labor, postpartum hemorrhage and maternal hospital stay after delivery. We used logistic regression to estimate odds ratios adjusted (aOR) for potential confounders.

**Results:**

In our sample, 67.3% of women had spontaneous labor and 32.7% were induced. More than half of the women received oxytocin during labor regardless of the mode of labor. The proportions of the composite outcome and of cesarean after onset of labor were higher in the induced group compared to the spontaneous group (26.1% vs 15.8%, p = 0.02 and 45.0% vs 27.6%, p<0.01, respectively). There were 9 uterine ruptures (2.6%) and this proportion was higher in the induced group compared to the spontaneous group, although this difference was not statistically significant (3.6% vs 2.2%, p = 0.48). After adjustment, labor induction was associated with higher risks of the composite outcome (aOR = 2.45, 95% CI: 1.29–4.65), cesarean after onset of labor (aOR = 2.06, 95% CI: 1.15–3.68) and maternal hospital stay after delivery ≥6 days (aOR = 6.20, 95% CI: 3.25–11.81). No association was found with postpartum hemorrhage.

**Conclusion:**

Labor induction after one prior cesarean was associated with a higher risk of adverse perinatal outcome. Nevertheless, the higher proportion of uterine rupture did not differ significantly from that in the spontaneous labor group.

## Introduction

Cesarean section rates increased worldwide from 12.1% in 2000 to 21.1% in 2015 [1]. Consequently, the number of pregnant women with previous cesarean deliveries has also increased. For instance, the proportion of pregnant women with a history of cesarean delivery increased in France from 8% to 11% between 2003 and 2016 [2, 3]. Cesarean births are an important concern because the presence of a uterine scar increases the risk of uterine rupture, which is associated with major risks of perinatal mortality and maternal and infant morbidity [4–8]. A previous cesarean delivery is also associated with higher risks of abnormal placentation [9, 10], hysterectomy [10, 11] and blood transfusion [10] for the subsequent pregnancies.

There are no adequately randomized controlled trials that have assessed the safety and effectiveness of methods of induction in women with a prior cesarean delivery [12]. Whether labor can be induced in women with a previous cesarean section is still debated, in particular in the case of an unfavorable cervix [13, 14]. Nevertheless, several observational studies have shown that labor induction in such cases is associated with a higher risk of uterine rupture when oxytocin is used compared with no oxytocin or spontaneous labor [5, 8, 15–18]. For induction with vaginal prostaglandin E2, the results are inconsistent: some studies found that prostaglandin E2 is associated with a higher risk of uterine rupture compared to other means of induction or spontaneous labor [5–7], while others did not [8, 19–21].

This study assessed perinatal outcomes associated with labor induction versus spontaneous labor among women with one previous cesarean delivery.

## Methods

### Study design and population

We conducted an observational retrospective study in one tertiary university hospital in France from January 1^st^, 2013 to October 26^th^, 2016. Data were obtained from the computerized database of the maternity unit recording all live births and stillbirths at 22 weeks of gestational age (GA) or more. This database includes information on maternal sociodemographic characteristics, medical and obstetrical history, pregnancy complications, delivery and infant outcomes at birth, as well as surgical reports.

This observational study used anonymized data from medical records and was approved by the CNIL (Commission Nationale de l’Informatique et des Libertés) under the notification number 2020 0117175609.

The study population included women with a history of one cesarean delivery and a singleton live fetus in cephalic presentation born at 37 weeks GA or more. Exclusion criteria were: preterm births <37 weeks GA, multiple pregnancies, stillbirths and terminations of pregnancy, non-cephalic presentation, no history of cesarean delivery, a history of uterine surgery. Women with an elective cesarean delivery for the current pregnancy were also excluded.

### Definition of outcomes

The primary outcome was a composite criterion of adverse perinatal outcomes including any of the following: uterine rupture, Apgar score <7 at 5 minutes and neonatal resuscitation in the delivery room or admission to a neonatal unit. The diagnosis of uterine rupture was based on surgical reports and defined as a complete disruption of the prior uterine scar including the peritoneum. Neonatal resuscitation was defined as oxygen administration or intubation in the delivery room. At the time of the study period, cord blood pH and lactate were determined only in cases of risk of fetal distress that included all emergency situations (cesarean section after onset of labor, placental abruption, uterine rupture, vasa previa, etc.). Because these tests were not systematically performed, we did not include cord blood pH and lactate in the composite criteria.

The secondary outcomes included cesarean section after onset of labor, postpartum hemorrhage and duration of maternal stay after delivery above 6 days. Based on French guidelines, postpartum hemorrhage was defined as a blood loss of at least 500 mL estimated by clinicians regardless of the mode of delivery [22]. We considered maternal hospital stay after delivery ≥6 days as an adverse outcome because the average length of hospital stay after a cesarean section in France in 2010 was 5.4 days [2].

### Covariables

Maternal characteristics included maternal age, parity, body mass index (BMI) before pregnancy and geographical origin. We also reported information on previous deliveries and current pregnancy complications. Neonatal characteristics included gestational age at delivery, birthweight and the infant’s sex. Birthweight was expressed as a continuous variable and in percentiles defined using an intrauterine growth reference adapted to the French population [23]. In France, gestational age is determined from the crown-rump length at the first ultrasound scan between 11^+0^ and 13^+6^ weeks [24].

Indications for labor induction were categorized into 5 classes: prolonged pregnancy, premature rupture of membranes (PROM), maternal medical cause (hypertensive diseases, diabetes, etc.), fetal cause (antenatal suspicion of growth restriction or macrosomia) and other indications. Indications for cesarean section after onset of labor were classified as follows: failure to progress, non-reassuring fetal monitoring, maternal cause and other indications. We also reported information on obstetrical management during labor: the type of analgesia, the use of intravenous oxytocin for labor augmentation and the method used for the induction of labor. In the maternity unit, the protocol for induction of labor in women who had one previous cesarean section and a favorable cervix (Bishop score ≥6) consisted of an amniotomy, secondarily associated with intravenous oxytocin. In contrast, vaginal prostaglandin E2 was used in women with an unfavorable cervix (Bishop score <6). Prostaglandin was kept for up to 24 hours with regular fetal heart monitoring. In the case of uterine contractions with cervical modifications or a favorable cervix, labor was continued with an amniotomy associated, if necessary, with oxytocin. A cesarean section was performed if the cervix was unchanged or unfavorable after 24 hours with vaginal prostaglandin E2. At the time of the study period, the Foley catheter and the double-balloon catheter were not used in the maternity unit.

### Statistical analysis

Maternal, pregnancy and neonatal characteristics as well as obstetrical management were compared between women with spontaneous labor and those with induced labor in univariate analysis using Student’s t-tests for continuous variables and chi-squared or Fisher exact tests for categorical variables. Statistical significance was defined for a p-value <0.05. The association between mode of labor and perinatal outcomes was assessed using a multivariable logistic regression to estimate odds ratios adjusted for potential confounding factors (aOR). We used directed acyclic graphs (DAGs) in order to select co-variables included in the multivariable models based on the literature and clinical assumptions. Interactions between labor induction and several co-variables (parity, pregnancy complications) were tested and were not significant. Because of the small number of uterine ruptures and neonates with a low Apgar score, we did not perform multivariable analysis separately for these two outcomes. However, these outcomes were included in the composite criteria of adverse perinatal outcomes. Finally, we described clinical characteristics and neonatal outcomes including cord blood pH and lactate related to cases with uterine rupture. Analyses were performed using Stata 13.0 software (StataCorp LP, College Station, TX, USA).

## Results

**Fig 1:**
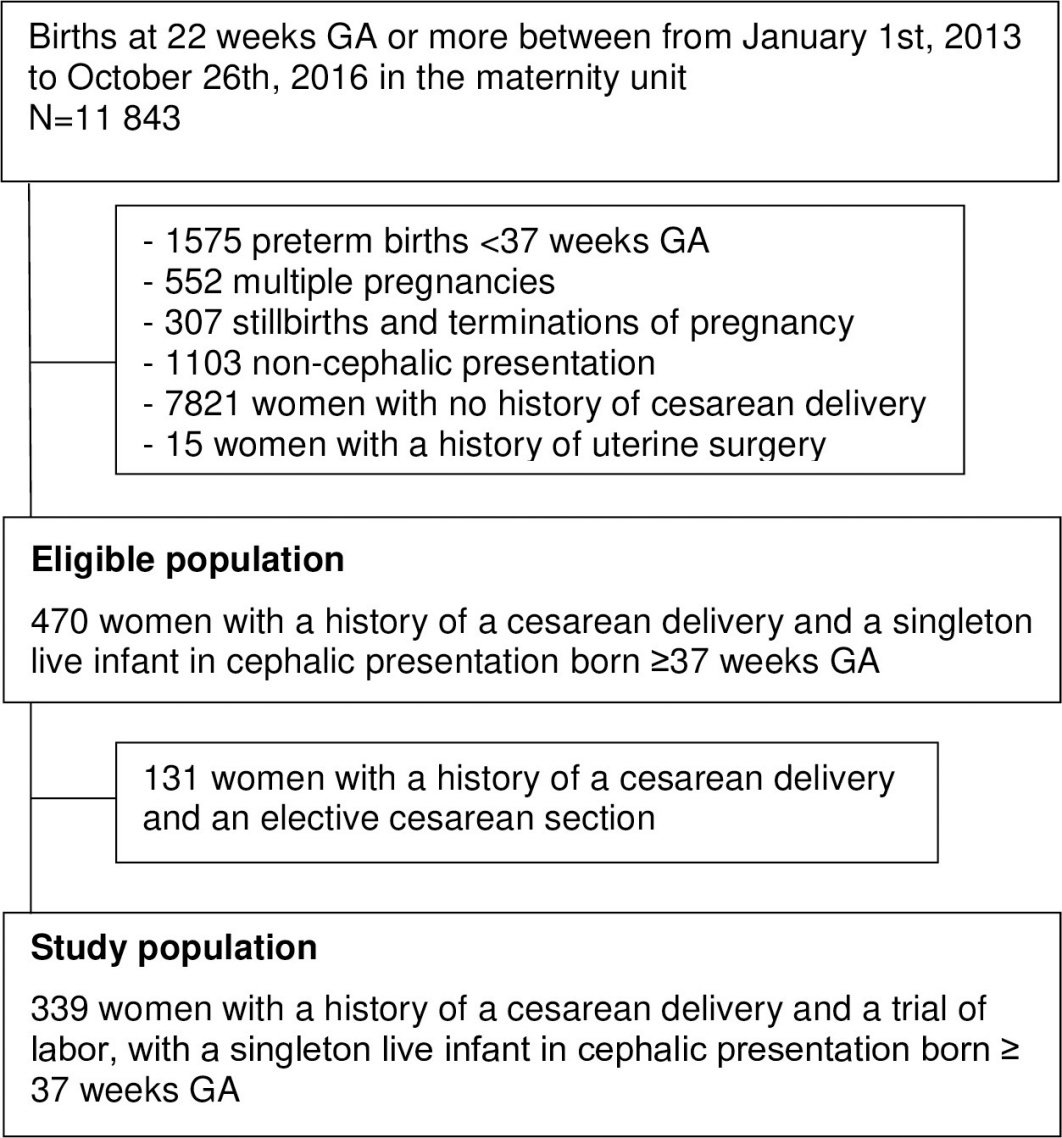
Flow chart.

Among the 11,843 births at 22 weeks GA or more recorded during the study period, 339 women had a history of one cesarean delivery and a singleton live fetus in cephalic presentation born at 37 weeks GA or more (Fig 1). Among the 339 women, 67.3% had spontaneous labor and 32.7% were induced. Maternal pre-pregnancy BMI was higher in the group of women with labor induction compared to women with spontaneous labor (26.1 kg/m^2^ vs. 24.3 kg/m^2^, p<0.01). (Table 1) None of the other maternal characteristics differed between the groups. More than half of the women received oxytocin during labor regardless of the mode of onset of labor.

**Table 1:** Maternal, obstetrical and neonatal characteristics according to mode of onset of labor.

	Total	Spontaneous labor	Induction of labor	p-value
	n (%) or mean ± sd	n (%) or mean ± sd	n (%) or mean ± sd	
Total	339 (100)	228 (67.3)	111 (32.7)	
**Maternal characteristics**				
Maternal age (years)	33.0 ± 4.4	33.0 ± 4.4	33.1 ± 4.6	0.89
Parity	1.3 ± 0.7	1.3 ± 0.7	1.4 ± 0.8	0.43
Pre-pregnancy BMI (kg/m^2^)	24.8 ± 5.2	24.3 ± 5.0	26.1 ± 5.5	<0.01
Geographical origin				
France	167 (49.3)	113 (49.6)	54 (48.6)	0.57
Europe	22 (6.5)	16 (7.0)	6 (5.4)	
Africa	121 (35.7)	77 (33.8)	44 (39.6)	
Other countries	29 (8.5)	22 (9.6)	7 (6.3)	
**Obstetrical history**				
Previous vaginal delivery	74 (21.8)	50 (21.9)	24 (21.6)	0.95
Indication of previous cesarean delivery				
Failure to progress	78 (23.0)	59 (25.9)	19 (17.1)	0.31
Non-reassuring fetal status	150 (44.2)	95 (41.7)	55 (49.5)	
Maternal indication	26 (7.7)	15 (6.6)	11 (9.9)	
Breech presentation	37 (10.9)	26 (11.4)	11 (9.9)	
Other	48 (14.2)	33 (14.5)	15 (13.5)	
Interval between cesarean section and actual delivery (months)	54.6 ± 36.7	53.2 ± 34.5	57.6 ± 40.9	0.31
Pregnancy complications				
None	206 (60.8)	156 (68.4)	50 (45.0)	<0.01^a^
Diabetes	63 (18.6)	28 (12.3)	35 (31.5)	
Hypertensive diseases	10 (2.9)	4 (1.7)	6 (5.4)	
Antenatal suspicion of FGR or macrosomia	24 (7.1)	14 (6.1)	10 (9.0)	
Other	36 (10.6)	26 (11.4)	10 (9.0)	
**Obstetrical management**				
Artificial rupture of membranes	164 (48.4)	108 (47.4)	56 (50.4)	0.59
Oxytocin use	187 (55.5)	123 (54.4)	64 (57.7)	0.58
Type of analgesia				
Epidural analgesia	312 (92.0)	215 (94.3)	97 (87.4)	<0.01^a^
Spinal anesthesia	16 (4.7)	5 (2.2)	11 (9.9)	
Other	4 (1.2)	2 (0.9)	2 (1.8)	
None	7 (2.1)	6 (2.6)	1 (0.9)	
**Neonatal characteristics**				
Gestational age at birth (weeks)				
37–38	62 (18.3)	37 (16.2)	25 (22.5)	<0.01
39–40	206 (60.8)	159 (69.7)	47 (42.3)	
41+	71 (20.9)	32 (14.0)	39 (35.1)	
Birthweight				
<10^th^ percentile^b^	34 (10.0)	22 (9.6)	12 (10.8)	0.88
10-90^th^ percentile^b^	280 (82.6)	190 (83.3)	90 (81.1)	
90^th^ percentile^b^	25 (7.4)	16 (7.0)	9 (8.1)	
Male sex	170 (50.1)	112 (49.1)	58 (52.2)	0.59

^a^ Fisher’s exact test

^b^ Based on an intrauterine growth curve adapted to the French population for gestational age and sex.

Among the 111 women who had induction of labor, the primary indication was maternal medical causes (34.2%) followed by prolonged pregnancy (26.1%). (Table 2) Labor was mainly induced with prostaglandin E2 (41.8%), then with both prostaglandin E2 and oxytocin (31.8%) and finally with oxytocin only (26.4%).

**Table 2:** Indications and methods used among women with induced labor.

	Induction of labor
	n (%)
Total N = 111	
Indications of induction of labor	
Maternal indications	38 (34.2)
Prolonged pregnancy	29 (26.2)
PROM	17 (15.3)
Fetal indications	10 (9.0)
Other	17 (15.3)
Induction with	
Prostaglandin E2 alone	46 (41.8)
Prostaglandin E2 + oxytocin	35 (31.8)
Oxytocin alone	29 (26.4)

Maternal and neonatal outcomes are compared according to the mode of onset of labor in Table 3. The rates of the composite adverse perinatal outcome and cesarean section after onset of labor were higher in women with labor induction compared to those with spontaneous labor (respectively, 26.1% vs. 15.8%, p = 0.02 and 45.0% vs. 27.6%, p<0.01). Overall, there were 9 uterine ruptures: 2.2% in the spontaneous group and 3.6% in the induced group (p = 0.48). There was a higher proportion of women who stayed more than 6 days after delivery in the induced group (39.1% vs. 9.0%, p<0.01). No difference was found between groups for postpartum hemorrhage. For neonatal outcomes, resuscitation in the delivery room was more frequent in infants born to mothers who were induced than in mothers with spontaneous labor (18.9% vs 9.6%, p = 0.02) and there was a non-significant trend to a higher rate of admission to a neonatal unit (14.4% vs. 8.3%, p = 0.08).

**Table 3:** Maternal and neonatal outcomes according to the mode of onset of labor.

	Total	Spontaneous labor	Induction of labor	p-value
	n (%)	n (%)	n (%)	
Total	339 (100)	228 (67.3)	111 (32.7)	
**Maternal outcomes**				
Cesarean section after onset of labor	113 (33.3)	63 (27.6)	50 (45.0)	<0.01
Uterine rupture	9 (2.6)	5 (2.2)	4 (3.6)	0.48^b^
Postpartum hemorrhage	32 (9.4)	24 (10.5)	8 (7.2)	0.33
Maternal hospital stay ≥6 days	63 (18.9)	20 (9.0)	43 (39.1)	<0.01
**Neonatal outcomes**				
Apgar score <7 at 5 minutes	4 (1.2)	3 (1.3)	1 (0.9)	1^b^
Resuscitation	43 (12.7)	22 (9.6)	21 (18.9)	0.02
Admission to a neonatal unit	35 (10.3)	19 (8.3)	16 (14.4)	0.08
Composite adverse perinatal outcome^a^	65 (19.2)	36 (15.8)	29 (26.1)	0.02

^a^ Includes uterine rupture, Apgar score <7 at 5 minutes, neonatal resuscitation in the delivery room or admission to a neonatal unit

^b^ Fisher’s exact test.

After adjustment for covariables, the risk of the composite adverse perinatal outcome was increased compared to women with spontaneous labor (aOR = 2.45, (95% confidence interval (CI): 1.29–4.65)). (Table 4) Induction was associated with higher risks of cesarean section (aOR = 2.06, 95% CI: 1.15–3.68) and maternal stay after delivery ≥6 days (aOR = 6.20, 95% CI: 3.25–11.81). No association was found with postpartum hemorrhage.

**Table 4:** Association between maternal and neonatal outcomes and induction of labor.

	Induction of labor	
	Crude OR (95% CI)	Adjusted OR (95% CI)
**Maternal outcomes**		
Cesarean section^a^	2.14 (1.33–3.44)	2.06 (1.15–3.68)
Postpartum hemorrhage^b^	0.66 (0.28–1.52)	0.66 (0.27–1.63)
Maternal hospital stay ≥6 days^c^	6.51 (3.58–11.84)	6.20 (3.25–11.81)
**Neonatal outcomes**		
Resuscitation^d^	2.18 (1.14–4.17)	2.49 (1.25–4.97)
Admission to a neonatal unit^e^	1.85 (0.91–3.76)	2.19 (1.03–4.64)
Composite adverse perinatal outcomes^e^	1.89 (1.08–3.28)	2.45 (1.29–4.65)

Reference group: spontaneous labor.

CI: confidence interval.

^a^ Adjusted for maternal age, parity, pre-pregnancy BMI, geographical origin, pregnancy complications, previous vaginal delivery, gestational age and birthweight.

^b^ Adjusted for maternal age, parity, pre-pregnancy BMI, epidural analgesia, oxytocin administration during labor, mode of delivery, episiotomy, duration of labor and birthweight.

^c^ Adjusted for maternal age, parity, pregnancy complications and geographical origin

^d^ Adjusted for birthweight, sex and gestational age.

^e^ Adjusted for maternal age, parity, pre-pregnancy BMI, geographical origin, previous vaginal delivery, oxytocin administration during labor, birthweight, sex and gestational age.

Table 5 reports information on the mode and duration of labor, gestational age at birth, drugs used to induce labor and the results of cord blood tests for the 9 uterine ruptures. Oxytocin was used in 6 cases (including 5 cases in women with spontaneous labor) and rupture of membranes was artificial in 7 cases. Three infants had a cord blood pH <7.15 and were transferred to a neonatal unit.

**Table 5:** Characteristics of the nine cases with uterine rupture.

	Labor and delivery characteristics				Neonatal outcomes		
Case	Type of induction	Gestational age at birth	Drugs used	Duration of labor (hours)	Cord blood pH	Cord blood lactate	Admission to a neonatal unit
1	Spontaneous	40+3	Oxytocin and ARM	4	6.99	8.6	No
2	Spontaneous	40+5	Oxytocin and ARM	10	7.29	4.2	No
3	Spontaneous	40+4	Oxytocin	4.5	7.16	8.7	No
4	Spontaneous	40+0	Oxytocin and ARM	8	7.19	5.9	No
5	Spontaneous	40+6	Oxytocin and ARM	5	7.18	3.1	No
6	Induction	40	PGE2	3	7.3	4.0	Yes
7	Induction	38+1	PGE2 and ARM	1	6.98	12.0	Yes
8	Induction	40+5	PGE2 and ARM	6.5	7.28	4.7	No
9	Induction	39+5	PGE2 and then oxytocin and ARM	2	7.13	7.7	Yes

ARM: artificial rupture of membranes; PGE2: prostaglandin E2.

## Discussion

### Main findings

In our maternity unit, one-third of women with a prior cesarean birth and a trial of labor were induced. Uterine rupture was seen in 2.6% of cases and this proportion was higher in the induced group compared to the spontaneous group, although this difference did not reach significance. Oxytocin was administered to more than half of the women. Labor induction was associated with higher risks of the composite adverse perinatal outcome, cesarean section and longer maternal stay after delivery compared to spontaneous labor.

### Limitations and strengths

Our study had several limitations. As we used an observational design, we were not able to establish a causal effect between mode of labor and observed perinatal outcomes. Our study was also retrospective, leading to a lower quality of data compared to a prospective study. However, adverse maternal outcomes as uterine rupture are rare and therefore many studies of the association between mode of labor and pregnancy outcomes in women with previous cesarean birth have also used a retrospective design [6, 8, 18, 19, 25–28]. In addition, the size of our sample was too small to compare methods of induction and therefore our analysis was performed in women who had induced labor regardless of the method used, as in other studies [19]. The sample size was also not sufficient to detect differences in outcomes such as uterine rupture, however, this outcome was analyzed using a composite criterion of adverse perinatal outcomes. Finally, the results of our single-center study cannot be extrapolated to medical practices in France. Nevertheless, we retrospectively reviewed all surgical reports in medical records while several previous studies included uterine rupture based on a diagnosis code using hospital discharge databases [15, 26].

### Interpretation

We found that labor induction was associated with an increased risk of cesarean section compared to spontaneous labor and this result is concordant with previous reports. In a large prospective study in the United States, labor induction was associated with a lower success rate of vaginal birth after a trial of labor [29]. One retrospective study also found a higher risk of cesarean section for women with a trial of labor compared to spontaneous labor [19]. In contrast, in a population of low-risk nulliparous women, a recent multicenter randomized trial found a lower risk of cesarean section after induction of labor [30] and this confirms that a first cesarean section has an impact on mode of delivery.

In previous retrospective population-based and single center studies, rates of uterine rupture were between 0.5% and 2% [5, 8, 17, 26]. In our series, this rate was 2.6% and was also high among women with spontaneous labor (2.2%). Several observational studies found an increased risk of uterine rupture related to labor induction after a prior cesarean birth [6, 8, 31], while other studies did not [19, 20]. In our study, almost one-third of women in the induced group received both prostaglandin and oxytocin; oxytocin was frequently used during labor, regardless of mode of onset of labor, and this practice may have an effect on the risk of uterine rupture even in women with spontaneous labor. A large prospective study using the National Institute of Child Health and Human Development Maternal–Fetal Medicine Units Network showed that the risk of uterine rupture increased with the use of oxytocin alone and increased more with the use of prostaglandin with or without oxytocin [32]. A nested-control study also found that a maximum dose of oxytocin above 20 mU/min was associated with a four-fold increased risk of uterine rupture [18]. Unfortunately, this information was not available in our study.

We found that labor induction was related to a ≥6-day maternal hospital stay after delivery. This result may be related to the higher proportion of cesarean section in the induced group as cesarean sections result in a longer hospital stay compared to vaginal deliveries. There was also a trend to a higher proportion of pregnancy complications and admission to a neonatal unit in the induced group and this could impact the duration of maternal stay after delivery. We did not find an association between labor induction and the risk of postpartum hemorrhage. This result is not concordant with previous studies, which found a higher risk of postpartum hemorrhage related to labor induction [33]. Many other factors such as duration of the second stage of labor could influence these results and were not investigated in our study.

After one previous cesarean birth, all professional societies recommend an assessment of individual risks to select candidates for a trial of labor [13, 34–37]. The selection of eligible women for a trial of labor after one previous cesarean birth would be relevant to the assessment of benefits and harms associated with the mode of birth. Compared to an elective repeat cesarean section, a trial of labor increases the risk of uterine rupture and is associated with a higher risk of neonatal hypoxic–ischemic encephalopathy [5, 7, 32]. In contrast, it is also important to avoid elective cesarean section because this increases the risk of maternal death and neonatal respiratory morbidity compared to vaginal delivery [38, 39]. However, a study using the 2010 French Perinatal Survey showed that 42% of women eligible for a trial of labor had an elective repeat cesarean delivery [40].

Prostaglandin E1 (i.e. misoprostol) was found to be associated with an increased risk of uterine rupture and there is a consensus not to use it in women with one previous cesarean delivery [13, 34, 41, 42]. In contrast, professional societies have not contraindicated the use of prostaglandin E2, but have recommended avoidance of its use in women with prior cesarean delivery [13, 34–36] in favor of the use of a Foley catheter, which seems to be associated with a lower risk of uterine rupture compared to induction with prostaglandins [6, 43]. However, a recent Cochrane review reported that randomized controlled studies of birth outcomes among women with prior cesarean section were underpowered to detect significant differences between methods of induction [12]. Large studies assessing the use of the Foley catheter to induce labor in women with a prior cesarean delivery are needed.

## Conclusion

Labor induction among women with a previous cesarean delivery was associated with increased risk of adverse perinatal outcomes. We estimated that the risk of uterine rupture was high, even in the case of spontaneous labor, highlighting that oxytocin administration during labor in women with a uterine scar should be used with caution and only if necessary.

## Supporting information

S1 Dataset (XLSX)
